# Cultivating Value Co-Creation in Health System Research Comment on "Experience of Health Leadership in Partnering with University-Based Researchers in Canada – A Call to Re-imagine Research"

**DOI:** 10.34172/ijhpm.2020.35

**Published:** 2020-03-11

**Authors:** Tracey K. Bucknall, Alison M. Hutchinson

**Affiliations:** ^1^School of Nursing and Midwifery, Faculty of Health, Deakin University, Geelong, VIC, Australia.; ^2^Centre for Quality and Patient Safety Research - Alfred Health Partnership, Institute for Health Transformation, Melbourne, VIC, Australia.; ^3^Centre for Quality and Patient Safety Research - Monash Health Partnership, Institute for Health Transformation, Clayton, VIC, Australia.

**Keywords:** Research Partnership, Value Co-Creation, Collaboration, Health System Research, Decision-Making

## Abstract

Partnerships have various purposes and exist in many configurations. Although there has been a refocusing in health system research on forming strategic partnerships between researchers and knowledge users (KUs) to maximise the relevance and uptake of research in practice; research knowledge frequently fails to reach KUs nor impact the community served. Whilst there have been many attempts to engage KUs, researchers and decision-makers often promote a top down approach that has lacked insight into KUs’ specific needs and values. Bowen and colleagues uncovered a plethora of negative experiences from a group of Canadian health leaders involved in researcher partnerships. Their comments reflect their experiences seemingly at an earlier stage of a partnership so we were not surprised by their pessimism. However, our experience reflects an established research-health service partnership network where we collaborate and co-create for mutual benefit and with a shared purpose. The reason for its sustained success over several decades is the focus on co-creation of value between stakeholders. Reimagining must prioritise a paradigm shift towards value co-creation if partnerships are to create opportunities for innovation, productivity and impact.

Knowledge is a critical asset in society today; useful for innovation, performance improvement and competitive advantage. Yet the volume of information for knowledge users (KUs) is exponentially growing and recognised to be incredibly difficult to transfer, regardless of system and discipline.^1,2^ Rather than linear cause and effect relationships, a more contempory view is one of interrelated networks that influence and emerge in an unpredictable way.^3^ Literature on complex systems demonstrates that knowledge is socially constructed in a dynamic, interconnected and interdependent context.^4^ Individuals actively create new knowledge, solving problems, interpreting information through a lens of prior knowledge, while continuously using and revising knowledge. Knowledge transfer is not the direct transplant of an object from one person to the next but rather the creation of new knowledge for each person based on their interactions, values, context and experiences.^2^ From this view, we know that knowledge is constantly adapted and applied in different contexts to become new knowledge. Partnerships focus on creating new knowledge together to innovate, enhance productivity and have a positive impact.

Bowen et al^5^ report on the experiences of health leaders in partnering with university-based researchers in Canada. The participants’ experience entailed a role in research across a health region or in a research partnership. The sample of 19 included 11 KUs, and 1 hybrid KU and researcher; in addition to the latter, there were only four researchers included in the sample. The article reports few participant details; broad roles of participants are reported, but a lack of detail makes it difficult to determine what their roles actually involved. Their level of experience was not provided.

Both researchers and KUs were favourable towards collaborative research initiatives and were consistent in their perceptions about the benefits of researcher-KU collaboration. They identified benefits for the research itself as ensuring research is relevant, promoting research quality and increasing the likelihood of uptake of the findings. At an organisational level, among other benefits identified were improved decision-making and practice, increased likelihood of research use, and improved capability for using research to inform policy. Learning, improved job satisfaction and observing new ways of tackling problems were some of the benefits identified at the individual level. In terms of societal benefits, it was perceived that the conduct of research examining problems encountered in the health system also benefited society, and collaboration addressed a moral imperative to demonstrate accountability for the use of tax-payer dollars.

Despite the consistency in opinions and the positivity among researchers and KUs about the benefits of collaboration for research, participants identified a number of challenges to collaboration. Issues to do with time availability, resources and communication were identified. Additionally, KU participants expressed negativity towards the research community (for the conduct of researcher-driven research) and pessimism about researchers’ intents. Lack of clarity about roles was also identified by KUs as problematic. Failures of multiple systems were also reported as sources of frustration. Also in the Canadian context, Sibbald et al^6^ recently explored barriers and attitudes towards collaboration in relation to funding opportunities that required researchers and KU partnerships. They found that despite perceived differences in roles, both groups were very positive regarding their partnerships. Barriers identified included resource constraints and differences in involvement and contribution among members. Despite these perceived barriers, both groups believed the partnerships were sustainable and helped create impact.

Partnerships have been conceptualised as existing on a continuum that extends from networking through coordinating and cooperating to collaborating,^7^ depending on the roles of each partner. Himmelman^7^ describes these strategies in terms of their relationship to time, trust and turf. Networking is the mutually beneficial exchange of information, which requires little time, trust or sharing of resources. Coordinating involves mutually beneficial information exchange as well as a change in activities to achieve a shared purpose. This strategy requires more time and trust, without the requirement for sharing of resources. Cooperating refers to mutually beneficial information exchange, change in activities and resource sharing to achieve a shared purpose; requiring substantial time, trust and resource sharing. Collaborating is the mutually beneficial exchange of information, change in activities and sharing of resources, combined with commitment to enhance capacity to achieve a shared purpose. This strategy requires greater levels of time, trust and resource sharing; with this risk comes sharing of risks and rewards.^7^ We consider co-creation to be an extension to the continuum, beyond collaboration. Similarly, co-creation requires a long term commitment to partnership as well as the development of capability ecosystems across sectors. Co-creation of knowledge involves greater stakeholder interaction to foster creativity, productivity and value.^8^

The experiences described by participants of Bowen and colleagues’^5^ study suggest they are functioning at an earlier stage of a partnership or collaboration continuum, reflective of disciplinary and sectorial silos, with a perception of research as an object to be transferred, exhibiting a lack of awareness of shared problems and absence or lack of shared values. The notion that researchers develop products that will suit the needs of KUs across all levels, regardless of the integrity of the adaptation process suggests a level of naivety about the role, purpose and outcomes of research. These beliefs and experiences may be a consequence of collaborations that have lacked depth and authenticity. Bowen et al^5^ have summarised some key established principles and called for a re-imagining of research partnerships, a radical rethinking of preparedness of researchers, and positioning research within health services, funding research activities and infrastructure. This is not radical, new thinking. We work within a partnership network established for 20+ years, with researchers located within health services, supported by health service and university infrastructure. While we recognise our network is unique internationally, and we endorse Bowen and colleagues’^5^ call for positioning research in health services, we also acknowledge that to establish and sustain such a collaboration requires numerous elements for success.

For any partnerships to flourish it becomes clear that there are two important areas on which to focus to enable knowledge creation: the intellectual features of the activities and the contexts in which the communication takes place. Bowen et al,^5^ like much of the research to date, have highlighted the challenges with partnerships in capricious environments, however few in health services research examine the intellectual features that influence knowledge creation. What part do individual attributes and abilities play in creating and absorbing new knowledge? Felin and Hesterly^9^ argued that individuals and their actions make up collectives and organisations, and that greater attention should be placed on what takes place inside human heads. To understand organisational level outcomes, they argue individuals must be considered an antecedent. How important are the attributes of individuals collaborating in a partnership to enable them to value co-create? We argue that there are skills, roles and personalities more suited to health service partnerships than others. These are not limited to but include: a relevant clinical background to understand the context of care; being approachable; being open to conversations and ideas; being willing to explore new areas that may extend beyond personal research interests; and being willing to facilitate introductions to others to progress problem solving.

The *dynamic knowledge transfer capacity model* (DKTC) may offer health leaders a structure to consider in the formation of research partnerships. DKTC approaches knowledge transfer using system thinking.^2^ It does not consider research as a transferable object, but as a consequence of exchanges between individuals within systems with varying knowledge capabilities. DKTC highlights the two pre-conditions for knowledge transfer as need and the existing knowledge the system possesses. These preconditions are the mainstay of the model on which to build the knowledge transfer capacity of a system. Parent et al^2^ described four types of capacities required within a system to facilitate knowledge transfer. *Generative capacity* focuses on discovery through research and development, *disseminative capacity* focuses on diffusion through social and technological infrastructure, *absorptive capacity* focuses on knowledge application and integration with prior knowledge, a readiness to change and management support. Lastly, *adaptive/responsive capacity* focuses on renewal through continuous learning, critical thinking and feedback amongst stakeholders. In particular, this pragmatic model considers both the intellectual and contextual elements. It illustrates the interconnected and interdependent relations requisite in knowledge transfer. Indeed, Bowen et al identified intra-disciplinary, multi-level and multi-sectorial challenges. DKTC identifies potential asset gaps that are not limited to researcher and KU partnerships, but knowledge creation more generally.

Janamian and colleagues^10^ described a framework to achieve value co-creation in primary care services research. They succinctly offered four elements essential for value co-creation which are relevant to other health systems. These were:

1. Time, effort and resources are necessary for effective long-term partnership development. Regular engagement between stakeholders is required to build relationships, co-creating value to maximise utility and impact.
2. Establish leadership and governance to manage stakeholder relationships and facilitate engagement to be co-creative throughout the whole process.
3. Understand the differing needs and priorities of the stakeholders to ensure outcomes meet those needs.
4. Clarify expectations and obligations between stakeholders to align perspectives, ensure transparency and mitigate the risk of perceived exploitation.

The importance of research partnerships to ensure new knowledge has relevance and utility for KUs, and to improve the likelihood that knowledge is applied in practice, is increasingly recognised. The strength of partnerships for health systems research exist on a continuum, extending from networking through to collaborating and co-creating value for mutual benefit with shared purpose. We argue that* re-imagining* research must prioritise a paradigm shift towards value co-creation if partnerships are to create opportunities for innovation, productivity and positive impact.

## Ethical issues

Not applicable.

## Competing interests

Authors declare that they have no competing interests.

## Authors’ contributions

Both authors contributed to the conceptualisation of the paper, drafting and agreement on the final paper.

## Authors’ affiliations

^1^School of Nursing and Midwifery, Faculty of Health, Deakin University, Geelong, VIC, Australia. ^2^Centre for Quality and Patient Safety Research - Alfred Health Partnership, Institute for Health Transformation, Melbourne, VIC, Australia. ^3^Centre for Quality and Patient Safety Research - Monash Health Partnership, Institute for Health Transformation, Clayton, VIC, Australia.
